# Optimal Serotype Compositions for Pneumococcal Conjugate Vaccination under Serotype Replacement

**DOI:** 10.1371/journal.pcbi.1003477

**Published:** 2014-02-13

**Authors:** Markku Nurhonen, Kari Auranen

**Affiliations:** Department of Vaccination and Immune Protection, National Institute for Health and Welfare, Helsinki, Finland; Brigham and Women's Hospital, United States of America

## Abstract

Pneumococcal conjugate vaccination has proved highly effective in eliminating vaccine-type pneumococcal carriage and disease. However, the potential adverse effects of serotype replacement remain a major concern when implementing routine childhood pneumococcal conjugate vaccination programmes. Applying a concise predictive model, we present a ready-to-use quantitative tool to investigate the implications of serotype replacement on the net effectiveness of vaccination against invasive pneumococcal disease (IPD) and to guide in the selection of optimal vaccine serotype compositions. We utilise pre-vaccination data on pneumococcal carriage and IPD and assume partial or complete elimination of vaccine-type carriage, its replacement by non-vaccine-type carriage, and stable case-to-carrier ratios (probability of IPD per carriage episode). The model predicts that the post-vaccination IPD incidences in Finland for currently available vaccine serotype compositions can eventually decrease among the target age group of children <5 years of age by 75%. However, due to replacement through herd effects, the decrease among the older population is predicted to be much less (20–40%). We introduce a sequential algorithm for the search of optimal serotype compositions and assess the robustness of inferences to uncertainties in data and assumptions about carriage and IPD. The optimal serotype composition depends on the age group of interest and some serotypes may be highly beneficial vaccine types in one age category (e.g. 6B in children), while being disadvantageous in another. The net effectiveness will be improved only if the added serotype has a higher case-to-carrier ratio than the average case-to-carrier ratio of the current non-vaccine types and the degree of improvement in effectiveness depends on the carriage incidence of the serotype. The serotype compositions of currently available pneumococcal vaccines are not optimal and the effectiveness of vaccination in the population at large could be improved by including new serotypes in the vaccine (e.g. 22 and 9N).

## Author Summary

The bacterial pathogen Streptococcus pneumoniae (pneumococcus) is a major contributor to child mortality worldwide. Hence, effective pneumococcal vaccination programmes are globally among the most cost-effective public health interventions. Three different conjugate vaccine compositions, targeting 7, 10 or 13 pneumococcal serotypes, have been used in infant vaccination programmes. The use of these vaccines has both decreased the disease burden and changed the patterns of pneumococcal carriage in locations where they have been in use. However, due to serotype replacement, where the lost vaccine serotype carriage is replaced by carriage of the non-vaccine serotypes, the net effect of vaccination on the disease burden has generally been milder than expected. Here, we apply a concise model for serotype replacement and present a ready-to-use tool for the prediction of patterns in post-vaccination pneumococcal incidence of carriage and invasive disease. We introduce a sequential algorithm for the identification of the most optimal additional serotypes to current vaccine formulations and demonstrate how differences in the invasiveness across serotypes imply that the disease incidence may either decrease or increase after vaccination. The methods we outline have direct relevance in decision making while reviewing the performance of the current pneumococcal vaccination programmes.

## Introduction

The bacterial pathogen *Streptococcus pneumoniae* (the pneumococcus) is a major cause of morbidity and mortality worldwide. Pneumococcal conjugate vaccines (PCV) were introduced over a decade ago and have proved highly effective in eliminating vaccine-type (VT) pneumococcal carriage and invasive disease (IPD) in countries where PCV has been included in the infant immunisation programme [1]–[3]. So far, three different PCVs have been in use: PCV7 (with vaccine serotypes 4, 14, 6B, 9V, 18C, 19F and 23F), PCV10 (additional serotypes 1, 5 and 7F) and PCV13 (additional serotypes 3, 6A and 19A). Despite the successes of PCVs against their respective vaccine types, the overall public health impact of pneumococcal conjugate vaccination remains less clear. In particular, the lost VT carriage has almost invariably been replaced by non-vaccine-type (NVT) carriage [4], [5]. Depending on the invasive potential of the serotypes involved, replacement in carriage leads to a varying degree of replacement in disease and may have undesirable implications on the overall pneumococcal disease burden in the population at large.

As population-wide changes in serotype-specific carriage and disease will not fully emerge until several years after the onset of a vaccination programme [6], mathematical models are indispensable in predicting the implications of serotype replacement on the use of pneumococcal vaccines. To date, dynamic models have been used in describing pneumococcal transmission, serotype competition as the mechanism of replacement and the net effectiveness of vaccination [7]–[9]. In addition, statistical models utilising serotype-specific carriage data and estimates of invasiveness (probability of disease per carriage episode) have been applied to predict post-vaccination disease patterns [10]–[12]. These and other authors have pointed out the importance of the invasiveness of replacing non-vaccine types when assessing the net effectiveness of PCV vaccination [3], [4], [13].

In this paper, we elaborate the above ideas to develop a concise model for serotype replacement and present a ready-to-use tool for the prediction of patterns in post-vaccination pneumococcal incidence of carriage and disease, based solely on pre-vaccination data on carriage and disease. For a given vaccine composition, corresponding either to a current or a prospective vaccine, we show in detail how the net effectiveness of vaccination under serotype replacement depends on the invasiveness of the vaccine types relative to that of the non-vaccine types. We demonstrate how differences in the invasiveness across serotypes imply that the disease incidence may either decrease or increase after vaccination and introduce a sequential algorithm for the identification of the most optimal additional serotypes to current vaccine formulations.

## Methods

### Data

The data on the prevalence of pneumococcal carriage in Finland originated from Syrjänen et al. [14] (<2 year olds), Palmu et al. [15] (4–5 year olds) and Leino et al. [16] (5+ year olds). The serotype distribution in carriage for under 5 years olds was based on data from <2 year old children in Finland [14] and for the rest of the population (5+ years) on data from England and Wales [4]. The UK data were available separately for the adolescent (5–19 year olds) and the adult (20+ year olds) populations. We combined these into a single set of serotype proportions corresponding to the 5+ age class by calculating the weighted averages of the proportions in the two age groups with weights 25% and 75%, respectively. The age- and serotype-specific annual average incidence of invasive pneumococcal disease (IPD) in 2000–2009 was retrieved from the National Infectious Disease Registry data (Finland).

Both carriage and IPD samples were utilised on the serogroup level, except for PCV7 serotypes, for which carriage data on the serotype level were available. IPD samples with serotype 6A were re-analysed to distinguish between serotypes 6A and 6C [17]. This information was not available from the carriage samples. The proportion of 6C carriage among 6A/C carriers was assumed to be one third in both age classes [4], [18], [19]. The sensitivity of the results on this assumption was explored by assuming alternative proportions (0% and 50%).

### The net effectiveness of vaccination

Our analysis of serotype replacement is based solely on age-specific serotype distributions in carriage and disease in the pre-vaccination era. Here, serotype distribution refers to the stationary (steady-state) distribution, assumed to be applicable in the pre-vaccination era or under a PCV programme with the current vaccine composition.

We first consider a single age stratum within which the proportion of vaccine-type (VT) carriage does not have notable trends with age. The pre-vaccination incidence of carriage and disease with serotype *i* are denoted by *c_i_* and *d_i_*, respectively. The case-to-carrier ratio *r_i_* is defined as the probability of disease per carriage episode, i.e. *r_i_* = *d_i_*/*c_i_*. At the aggregate level of all vaccine types, the corresponding quantities are
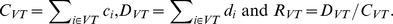



For the set of non-vaccine serotypes (NVT), the quantities *C_NVT_*_,_ *D_NVT_* and *R_NVT_* are defined similarly. We note that the aggregate case-to-carrier ratios are weighted averages of the respective serotype-specific case-to-carrier ratios, the weights being the carriage incidences. In the applications of this paper, disease is equivalent to invasive pneumococcal disease (IPD), but the proposed model is applicable to any disease outcome.

Our model of serotype replacement is built on two assumptions regarding the new steady-state after vaccination:

(A1) the relative serotype proportions among the non-vaccine types are not affected by vaccination (proportionality assumption);

(A2) the case-to-carrier ratios remain at their pre-vaccination levels.

It follows from assumptions (1) and (2) that the case-to-carrier ratios remain at their pre-vaccination values also for the aggregate VT and NVT sets.

**Figure 1:**
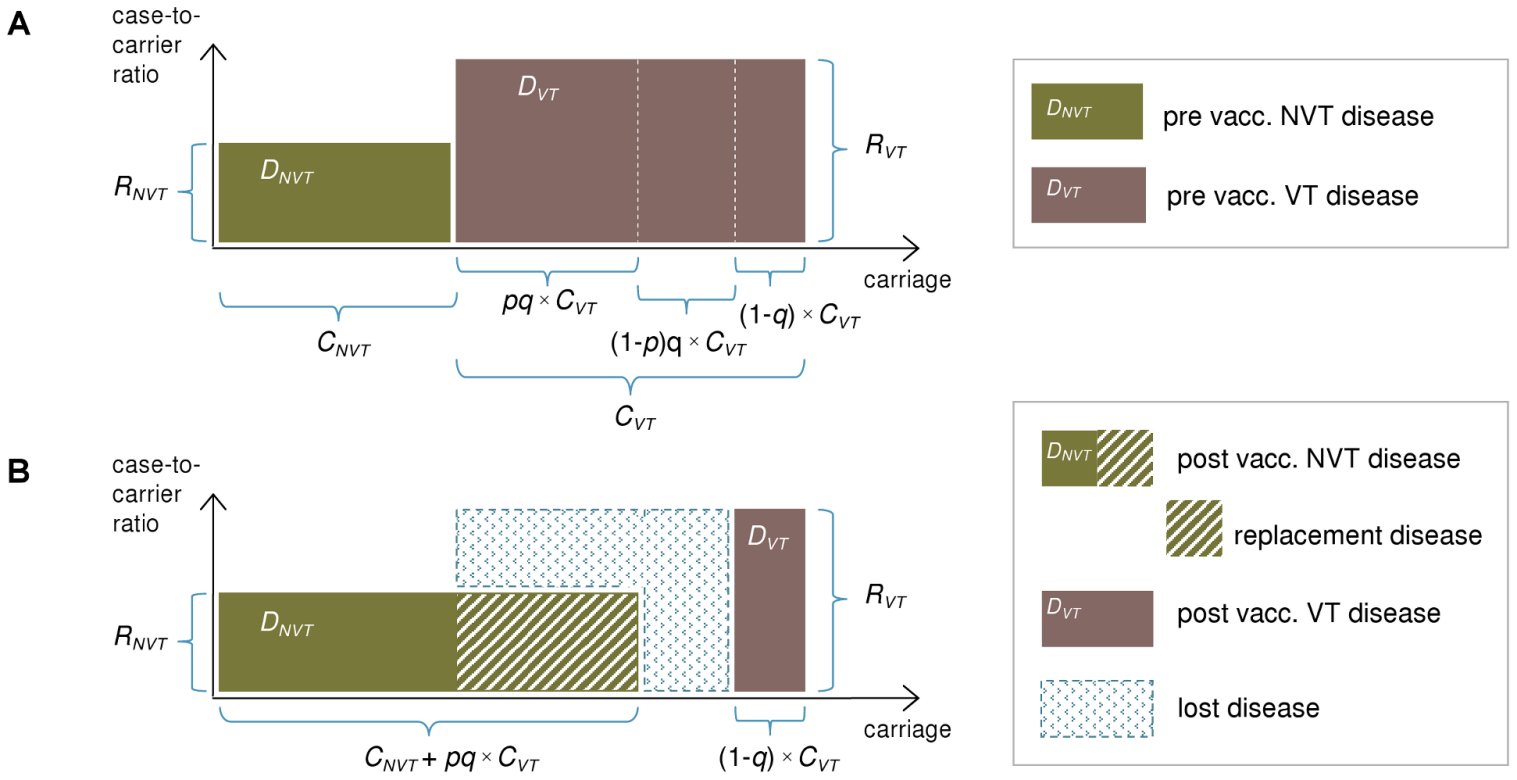
Illustration of the replacement model. The incidence of pneumococcal carriage (x-axis) and case-to-carrier ratios (y-axis) for vaccine serotypes (VT) and non-vaccine serotypes (NVT) before (panel A) and after vaccination (panel B). The incidences of disease (*D_NVT_* and *D_VT_*) are obtained by multiplication of the two quantities and correspond to the areas of the rectangles. VT carriage is divided into 3 segments corresponding to different effects of vaccination on population-level carriage: (i) VT elimination with replacement (proportion *pq* of VT carriage); (ii) VT elimination without replacement (proportion (1-*p*)*q* of VT carriage; and (iii) non-elimination (proportion (1-*q*) of VT carriage). Here *q* is the proportion of VT carriage eliminated by vaccination and *p* is the proportion of eliminated VT carriage replaced by NVT carriage. For notation, see text. The decrease in IPD incidence after vaccination is obtained as the difference between the eliminated VT disease and the replacing NVT disease. This is the combined area of the blue rectangles in panel (B).

Let *q* be the proportion of VT carriage eliminated by vaccination and *p* the proportion of eliminated VT carriage that is replaced by NVT carriage in the new steady state. Under assumptions (A1) and (A2), the disease incidence after vaccination is (for clarification, see Figure 1)
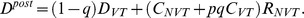



The reduction in the disease incidence is thus
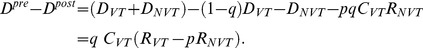
(1)


As *R_VT_* = *D_VT_*/*C_VT_* and *R_NVT_* = *D_NVT_*/*C_NVT_*, the following expression is equivalent to (1):
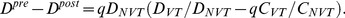
(2)


According to these, the disease incidence can either decrease or increase after vaccination. In particular, whether or not vaccination will be beneficial depends on the magnitude of the case-to-carrier ratio of the vaccine types compared to that of the non-vaccine types. According to equation (1), a reduction in the disease incidence requires the (average) VT case-to-carrier ratio (*R_VT_*) be larger than an (adjusted) NVT case-to-carrier ratio (p*R_NVT_). An alternative characterisation follows from (2), according to which a disease reduction requires the vaccine types possess a larger share of pre-vaccination disease than their (adjusted) share of pre-vaccination carriage. These characterisations are independent of the degree of elimination *q*. Of note, if the mean duration of carriage is the same for all serotypes, the above expressions are equally valid if carriage prevalences are used instead of the incidences *C_VT_* and *C_NVT_*.

#### Age-stratification

Age is a confounder in any analysis of replacement, because of its association with both the distribution of VT/NVT and the risk of disease (i.e. case-to-carrier ratios). Therefore, the analysis needs to be adjusted for age by appropriate stratification by age categories. If not adjusted, predictions of serotype replacement may be biased due to collapsed heterogeneous sub-strata.

## Search for optimal vaccine serotype compositions

Rewriting equation (2) for a single serotype *i* with *q* = 1, we obtain a criterion for the serotype whose addition to the vaccine would result in the largest decrease in the disease incidence. In particular, by substituting *D_VT_ = d_i_* (disease incidence of serotype *i*) and *D_NVT_ = D* – *d_i_*, where *D* is the total disease incidence, and likewise *C_VT_* = *c_i_* and *C_NVT_* = *C - c_i_*, equation (2) can be written as
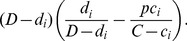
(3)


The optimal serotype is the one that maximises expression (3). Clearly, the best single vaccine type is neither necessarily the one with the highest pre-vaccination incidence of IPD nor the one with the highest case-to-carrier ratio. In fact, the optimal single serotype may not correspond to the highest value of either of these two quantities. Importantly, the optimal type may be different for different age groups. Furthermore, if *c_i_* is equal (or close) to 0, the decrease in IPD incidence (3) is equal (or close) to *d_i_*, i.e. the decrease is determined solely by the IPD of the serotype.

Equation (3) can be applied sequentially to find the optimal vaccine with a given number of serotypes. At each step the current vaccine composition is supplemented by the best single serotype among those not already included in the vaccine. This is repeated until a desired number of vaccine serotypes is reached. In most cases this procedure can be expected to lead either to the maximum possible reduction in IPD or at least to a reduction very close to the maximum.

Note that the optimal serotype composition is independent of the assumed level of VT elimination. If the elimination of VT carriage is expected to be incomplete (*q*<1) and proportion (1-*q*)*100% of VT carriage remains after vaccination, the projected incidences of carriage and IPD are obtained as weighted averages of the model projections assuming complete elimination and of the original incidences with weights *q* and *1-q*, respectively.

A program code implementing the tools proposed above, including instructions on how to use the code, is provided in File S1. The program is written in the R programming language, which is freely available.

## Uncertainty in carriage proportions

As small changes in the VT/NVT carriage proportions may result in notable shifts in projected IPD incidences, uncertainties in carriage data should be accounted for using sensitivity analysis. When assessing the net effectiveness of vaccination with a given vaccine composition, the effect of a change in the VT/NVT carriage proportions is obtained directly from (2). To investigate the robustness of the optimal serotype composition, we calculated the order of inclusion of individual serotypes in the optimal vaccine composition for a large number of sets of carriage proportions, which were generated from an uncertainty distribution. For more details, see File S2.

## Proportionality assumption (A1)

We investigated the similarity of pre- and post-vaccination serotype proportions for the non-vaccine types, based on published data from three different locations [4], [10], [19]. This analysis shows that the proportionality assumption (A1) is solid as a general rule (see Figure S1).

## Results

### Net effectiveness under different vaccine formulations

We applied the replacement model to the pre-vaccination IPD incidence and carriage prevalence data to predict the post-vaccination IPD incidence in Finland.

#### Replacement in IPD for PCV10 and PCV13

**Figure 2:**
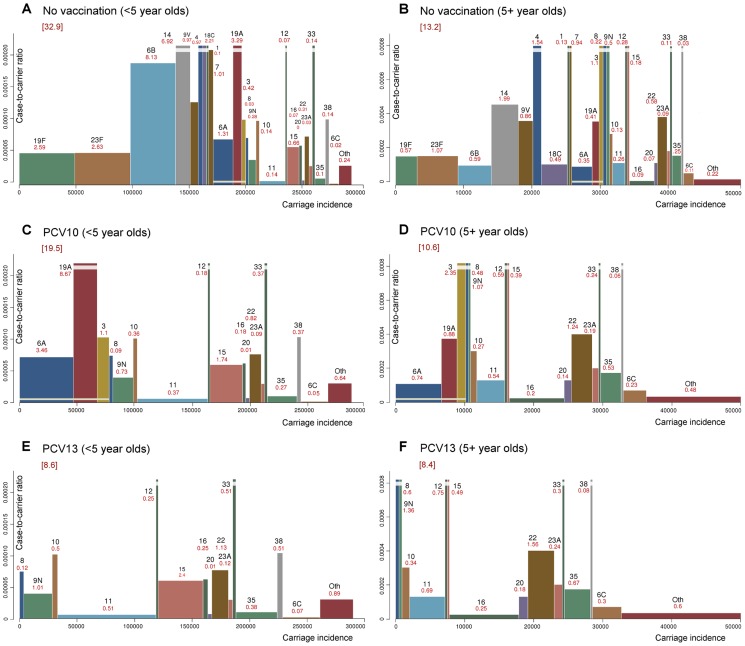
Projected IPD incidence in Finland under complete replacement in carriage. The effects of complete replacement in carriage for 2 vaccine formulations (PCV10 and PCV13) for under 5 year old children (panels A, C and E) and the rest of the population (panels B, D and F). The x-axes correspond to average annual incidences of pneumococcal carriage per 100,000 persons. The incidences are scaled from the prevalence data by dividing by the mean duration of carriage (1.5 months for under 5 olds and 1 month for the rest). The width of each rectangle corresponds to the average annual serotype-specific carriage incidence and the height (y-axes) to the case-to-carrier ratio. In the “no vaccination” panels (A and B) the area of each rectangle is the observed annual IPD incidence in Finland (average number of cases from 2000–2009) and in the other panels the area corresponds to the projected IPD incidence. The analysis pertains to serogroup level, except for the PCV7 serotypes. The observed (panels A and B) and projected (other panels) incidences of IPD are indicated in red under the serotype labels. The projected total IPD incidences are indicated under the PCV labels. In panels A and B, the PCV10 types are listed first and the 3 additional PCV13 types are indicated by a horizontal yellow stripe near the bottom of the bars in panels A–D. For clarity, some of the bars with high case-to-carrier ratios are truncated. These are indicated by horizontal white stripes near the top of the bars. Category “other” includes the following 19 serotypes or serogroups with small IPD incidences: 13, 17, 18BF, 19BCD, 2, 21, 24, 25, 27, 28, 29, 31, 34, 37, 39, 40, 41, 46, 9A.

Figure 2 illustrates the implications of serotype replacement under PCV10 or PCV13 programmes on the overall and serotype-specific incidence of IPD in Finland, when complete VT elimination and replacement are assumed (*p = q = *1). Each rectangle represents one individual serotype with the area equaling to its IPD incidence, obtained as product of carriage incidence (width) and case-to-carrier ratio (height). The serotype-specific case-to-carrier ratios fall broadly in two categories, the most invasive ones (e.g. 1, 4, 7, 12, 33) being largely the same in both age groups. After vaccination, the carriage and IPD incidences of non-vaccine types expand by a proportion corresponding to the lost VT carriage [assumption (A1)] whereas the case-to-carrier ratios remain at their pre-vaccination levels [assumption (A2)].

According to these predictions, PCV10 and PCV13 are highly effective in reducing the IPD incidence among the under 5 year olds. For example, use of PCV10 is expected to eventually reduce the IPD incidence in this age group by 40% and the use of PCV13 by 75%, i.e. from 99 to 59 and 25 annual cases, respectively, in a population of approximately 300,000 children. No clear candidates emerge as additional vaccine types to supplement PCV13. However, for the older population, the predicted reductions are clearly smaller (20% for PCV10 and 36% for PCV13) and neither of the two serotype compositions is optimal as inclusion of some of the commonly carried serotypes appears not to be beneficial. In particular, serotypes 19F and 6B have low case-to-carrier ratios as compared to the non-vaccine types and are thus good candidates for the set of replacing non-vaccine types in this age group.

The proportion of serotype 6C among 6A/C isolates from IPD was 0% in children and 24% in the 5+ age group. Because 6C has relatively low case-to-carrier ratios in both age categories, it is an ideal non-vaccine type. If the proportion 6C carriers among 6A/C carriers would be smaller than the assumed 33%, the predicted IPD incidence for any of the vaccine compositions including 6A, but not 6C, would be higher. The incidence would be 5–10% higher if no 6C carriage is assumed (Table S1). Furthermore, if serotype 6A is considered vaccine-related due to 6B ([18], see Figure S1), the predicted IPD incidences under PCV7 and PCV10 would be higher.

#### IPD reduction as a function of VT proportions in carriage and in IPD

**Figure 3:**
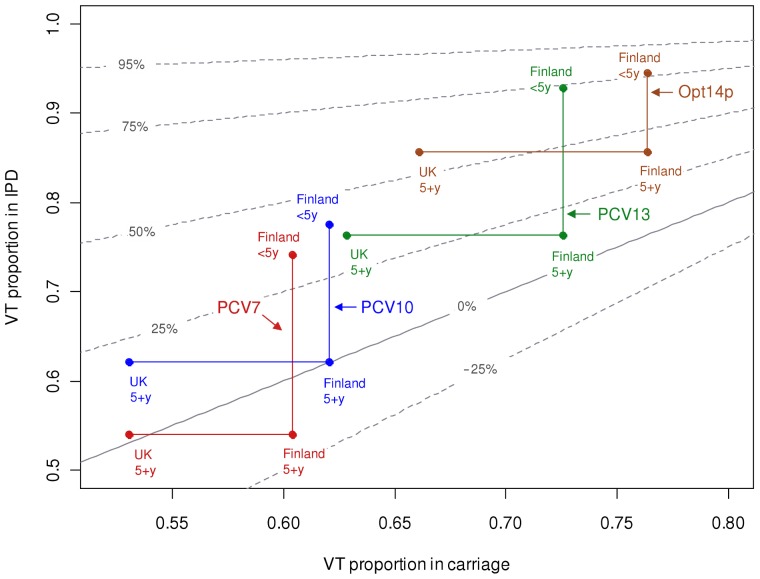
Predicted reduction in IPD incidence as a function of pre-vaccination proportions of VT carriage and disease. Each curve (drawn in grey colour) corresponds to all possible combinations of the pre-vaccination proportions of VT carriage (x-axis) and VT disease (y-axis) that lead to the indicated level of reduction in IPD incidence (95, 75, 50, 25, 0 and −25%). The results are shown for complete replacement in carriage. The locations of 12 combinations regarding the VT carriage and VT disease proportions are superimposed. These combinations pertain to 4 different vaccine formulations (PCV7, PCV10, PCV13, Opt14p) and two age groups (<5 or 5+ year olds); for the older age group, two alternative carriage proportions are presented. In each case, the Finnish IPD data were used. The 14-valent vaccine composition Opt14p refers to a hypothetical vaccine discussed in Figure 5. Scenarios pertaining to the same vaccine composition but different carriage data for adults are connected by horizontal lines. Scenarios pertaining to the same carriage data but different age groups are joined by vertical lines. For results under partial replacement in carriage, see Figure S2.

Figure 3 shows how the relative reduction in the disease incidence depends on the pre-vaccination proportions of VT carriage (shown on the horizontal axis) and VT disease (shown on the vertical axis). In general, the net effectiveness of vaccination is the better the larger VT proportion in IPD and the smaller the VT proportion in carriage are prior to vaccination. When PCV7, PCV10 and PCV13 are compared, increasing the valency of the vaccine increases the effectiveness of vaccination. Under the assumption of incomplete replacement, as the degree of replacement decreases, vaccination becomes more effective and the importance of VT disease proportion increases, eventually becoming the only determining factor under the no replacement assumption (see Figure S2).

Figure 3 can be used to study the sensitivity of predictions on the uncertainty regarding the pre-vaccination proportion of VT carriage or disease. For example, if the proportion (62%) for PCV10-VT carriage among the Finnish children (<5 years of age) is used for 5+ year old individuals, instead of that based on adult data from the UK (53%), the predicted relative reduction in IPD incidence under PCV10 among the 5+ age category will decrease from 19% to 0%.

### Optimal vaccine serotype compositions

#### The most beneficial single serotype

**Figure 4:**
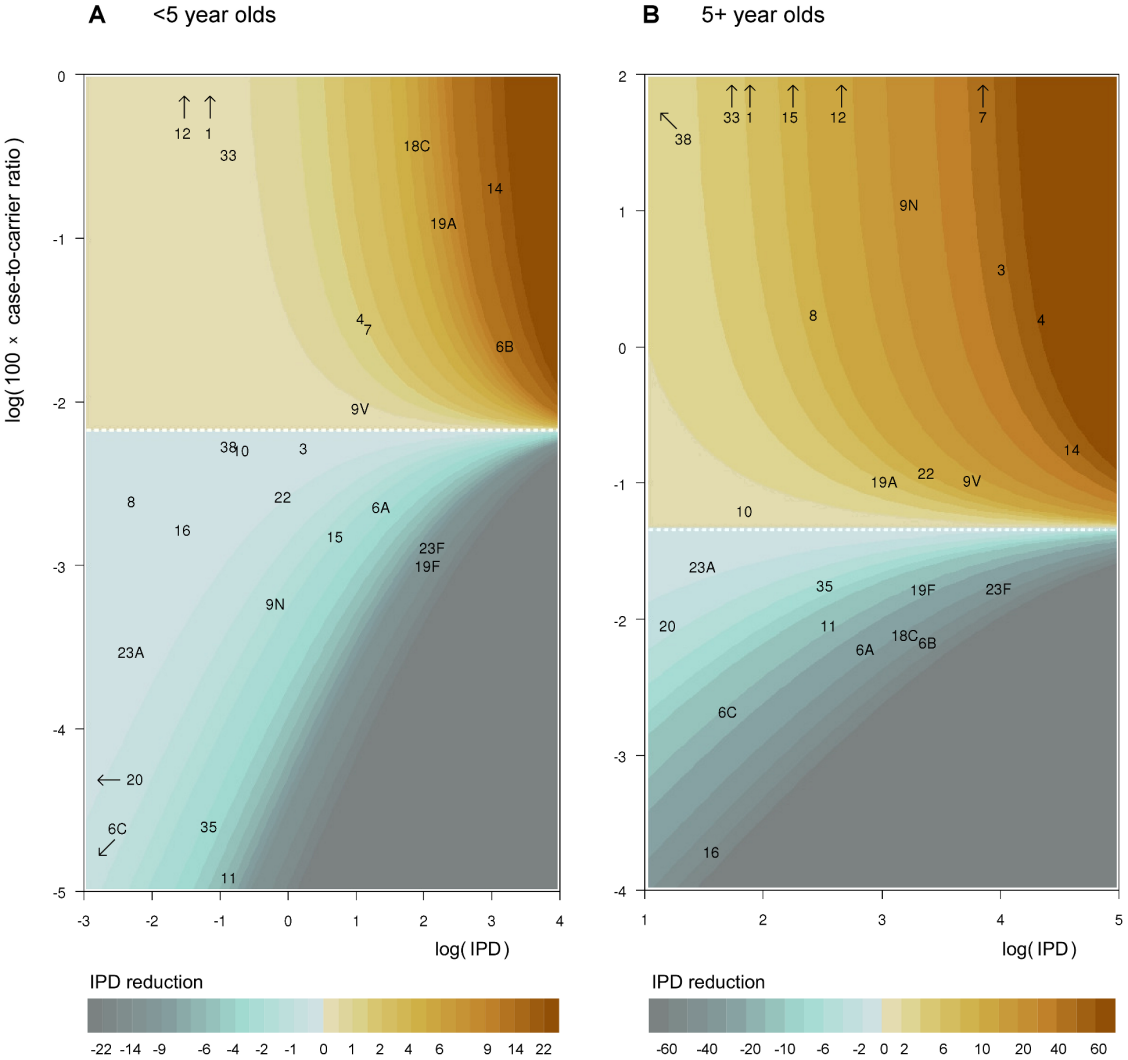
Effect on IPD of adding a single new vaccine type. Contour plots of the effect of a single vaccine type as a function of serotype-specific IPD incidence (horizontal axis) and case-to-carrier ratio (CCR, vertical axis) under the complete replacement model. Panel (A) corresponds to children under 5 years of age and panel (B) to the rest of the population. The predicted decrease in annual IPD incidence from the IPD incidence under no vaccination (99 and 658 for <5 and 5+ year olds, among populations of size 300,000 and 5,000,000, respectively) is indicated by colour codes. Interpretation of the colour codes in terms of decrease in IPD incidence, separately for each panel, is shown below the panels. The horizontal white dashed line corresponds to no effect. The axes are in log scale. Serotypes outside of the range of the plot due to a high case-to-carrier ratio or a low IPD incidence are indicated by arrows.

Figure 4 illustrates how the expected reduction in the overall IPD incidence due to a hypothetical vaccine with only one serotype depends on the serotype's pre-vaccination IPD incidence and case-to-carrier ratio [cf. equation (3)]. The white dashed line corresponds to the weighted average case-to-carrier ratio and serotypes above this line would be beneficial. Each of the serotypes 4, 7, 9V, and 14 would be beneficial among both under 5 year olds and the rest of the population. By contrast, serotypes 11 or 35 would increase the IPD incidence in both age groups. Serotype 3 would be beneficial mainly among the 5+ year category and 6B only among the <5 year category.

#### Optimal serotype compositions

**Figure 5:**
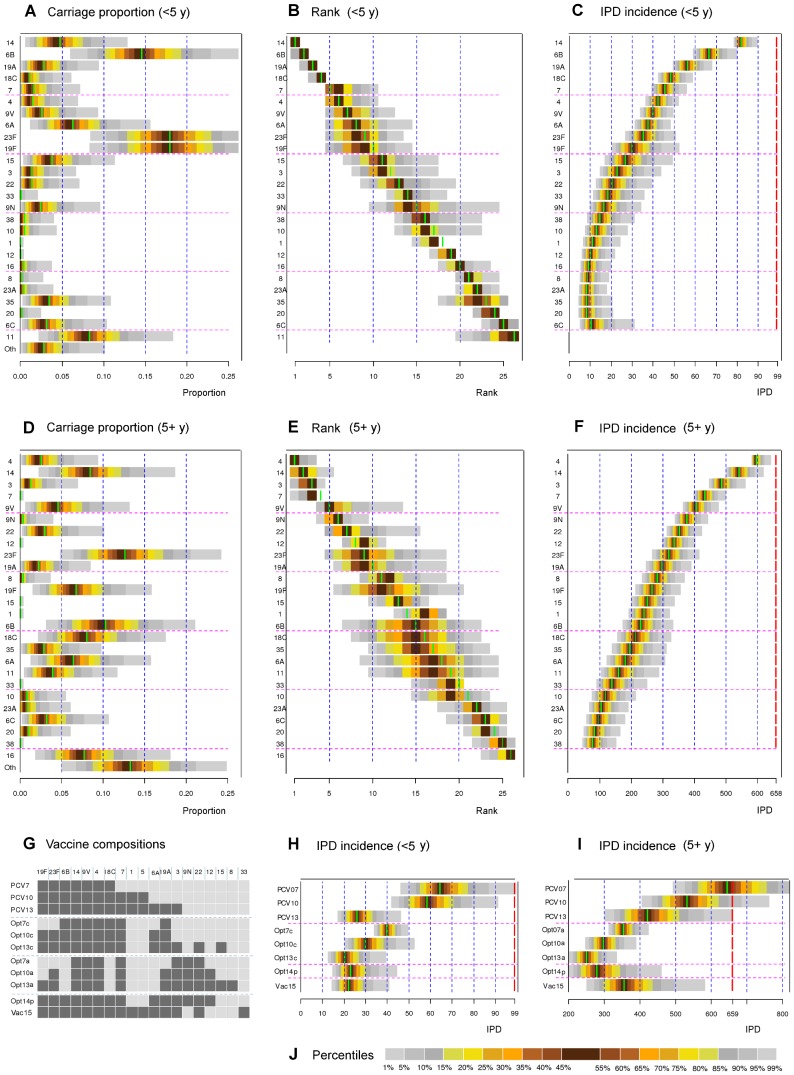
Optimal serotype compositions. (**A**) The observed serotype-specific proportions in carriage among children <5 years old (Finland) with uncertainty distribution (see File S2). The colours indicate percentiles of the distribution as shown in panel J. In each panel, the green bar corresponds to the result obtained using the observed proportion in carriage (see Figure 2). (**B**) Serotypes in the order of importance in the vaccine composition (from top to bottom) for children <5 years old. The order is based on sequentially adding a new serotype to the current composition so that the new serotype corresponds to the highest IPD reduction. In addition, uncertainty about each serotype's rank in the sequence is shown, based on the uncertainty in carriage data. As the ranks are integer numbers, some percentage points of the distributions are not shown. (**C**) Annual IPD incidence in <5 year olds (population of size 300,000) in Finland, corresponding to each sequential step. For example, the third horizontal bar for the <5 year olds means that adding serotype 19A to a bivalent vaccine with serotypes 14 and 6B is expected to lead to 57 annual IPD cases. The uncertainty in IPD predictions induced by uncertainty in the carriage data is shown. The dashed red line on the right corresponds to the annual IPD incidence under no vaccination (99). (**D**),(**E**),(**F**) As panels A–C, but for the population 5+ years of age (population size 5,000,000). The no vaccination IPD incidence is 658. (**G**) List of the 11 different vaccine compositions of interest: PCV7, PCV10, PCV13; the optimal (hypothetical) 7, 10 and 13-valent vaccines: Opt7c, Opt10c and Opt13c for <5 year of olds; Opt7a, Opt10a and Opt13a for 5+ years olds. In addition, Opt14p refers to a (hypothetical) vaccine composition optimised for the whole population and Vac15 to a possible future composition [22]. Dark square indicates inclusion in the corresponding vaccine composition. (**H**),(**I**) The projected IPD incidences for the vaccine compositions in panel G corresponding to perturbations of the serotype proportions as shown in panels A and D. (**J**) Colour codes corresponding to the percentiles of the distributions. For optimal serotype compositions under partial replacement in carriage, see Figure S3.

Applying criterion (3) sequentially provides an optimal order of introducing serotypes to a vaccine. Panel B in Figure 5 shows this order among the <5 year olds, taking into account uncertainty in carriage proportions (panel A). The corresponding IPD predictions are shown in panel C. Panels D, E and F in Figure 5 show the results in the 5+ population. In this age group, the decrease in the IPD incidence from the pre-vaccination level after each additional type is 5–15% for the 10 most important types and less thereafter. After 15 serotypes, the decrease is very small.

Irrespective of the age class, serotypes 14, 4, 9V and 7 are beneficial. Apart from these serotypes, however, the optimal serotypes depend on the age class of interest. Serotype 6B is beneficial only among children <5 years and serotype 3 only among the 5+ year olds. Among the <5 year olds, inclusion of the 7 most optimal serotypes results in a reduction of 60% in IPD. Similarly 13 most optimal types lead to a reduction of 80%. For the 5+ year old population the corresponding reductions are smaller (45% and 60%).

In addition, a composition of a 14-valent vaccine was identified so that the resulting reduction in IPD is no worse among under 5 five years olds than with the current PCV13 and at the same time substantial in the general population. This vaccine composition results in a 75% reduction among under 5 year olds and 40% among the rest of the population (Figure 5). Of note, the existing PCV13 vaccine formulation is close to optimal among the <5 year olds whereas among the 5+ year olds the net effectiveness of any of the PCV formulations (PCV7, PCV10 and PCV13) is less than optimal under the complete replacement model. Uncertainty in carriage data does not alter the relative importance of individual serotypes significantly but does affect the projected net effectiveness of vaccine compositions. In particular, for any of the current PCV formulations, there is an appreciable (PCV7) or small (PCV10, PCV13) predictive probability that the net effectiveness among adults is negative. Similarly, assuming less than complete replacement affects more the projected net effectiveness than the set of the most important serotypes (Figure S3).

## Discussion

We derived a simple expression for the expected net effectiveness of childhood vaccination against invasive pneumococcal disease (IPD) under serotype replacement. This expression depends only on the pre-vaccination incidences of vaccine-type (VT) and non-vaccine-type (NVT) carriage and disease. Our analysis explicates that vaccination will result in a notable reduction in the IPD incidence only if the average case-to-carrier ratio of the vaccine types clearly exceeds that of the non-vaccine types. In Finland, this would be expected to occur among children with any of the currently available PCV formulations. However, the same might not hold to the same extent in the general population not targeted by the vaccination, and our analysis indicates there are vaccine compositions with higher expected net effectiveness. These compositions are projected to be no worse than the current ones among children while clearly outperforming them in older age categories.

We formulated the expected net effectiveness of vaccination in terms of serotype-specific incidences of carriage and disease. Equivalently, one could use either of the two quantities together with the case-to-carrier ratios (i.e. disease incidences divided by carriage incidences) as any two of the three quantities determine the third one. Importantly, while beneficial vaccine serotypes can be identified using carriage and disease data, they are not necessarily those with the highest carriage incidence, disease incidence or case-to-carrier ratios. The net effectiveness will be improved only if the average VT case-to-carrier ratio is larger than the average NVT case-to-carrier ratio.

Furthermore, the above rule is not transparent, unless some serotype had the largest incidence of either carriage or disease and at the same the largest case-to-carrier ratio. In addition, a trivial rule applies in two special situations. First, if all serotypes have identical case-to-carrier ratios, as may be a good first approximation in case of pneumococcal otitis media, there is essentially no change in disease incidence. Second, if there is no replacement in carriage, the expected change in disease only depends on the pre-vaccination disease incidence.

Stratification of carriage and disease data by age is essential in the analysis of replacement. For example, an individual serotype included in a vaccine may decrease IPD in one age category while increasing it in another (cf. serotype 6B among <5 and 5+ year olds in Finland; Figure 2). If an optimal protection for the whole population is of interest, such opposite effects across different age categories may be partly compensated by the inclusion of a sufficient number of serotypes. In particular, while the analysis shows that among the <5 year olds in Finland PCV13 cannot be much improved, the adverse effect among adults of including 6B in the vaccine could be compensated by including additional serotypes (e.g. 9N, 12, 22).

In practice, evaluation of optimal PCV vaccination for the whole population should be based on a cost-effectiveness analysis that takes into account health benefits and costs in the vaccine target population as well as in the older cohorts. Of note, all of the serotype compositions we consider refer to an infant vaccination programme assuming an adequate level of coverage of vaccination to induce a substantial herd effect in the whole population.

The algebraic simplicity of our model is a direct consequence of the two key assumptions that neither the serotype proportions in carriage nor the invasiveness (case-to-carrier ratios) of the non-vaccine types are altered by vaccination. Examples of post-vaccination scenarios not covered by our model are a disproportionally large increase in carriage of a previously rare invasive type or an increase in invasiveness of a commonly carried type. Either of these scenarios would increase the IPD in excess of our model predictions. However, there is some empirical evidence in support of the key assumptions. In particular, our analysis confirmed the similarity in pre- and post-vaccination serotype proportions using four different datasets (Figure S1). The unchanged invasiveness of individual non-vaccine serotypes under vaccination is suggested by their relatively stable case-to carrier ratios in different populations pre-vaccination [3].

The pre- and post-vaccination incidences of carriage and disease involved in our method correspond to the respective stationary (steady-state) serotype distributions. These distributions can be characterised by the average annual serotype-specific incidences over a period where they do not manifest any systematic trends. The post-vaccination stationary distribution is typically achieved some years after the onset of a new infant vaccination programme [1], [2], [20]. Importantly, if post-vaccination data on IPD are available, the reliability of the model predictions can be monitored at an intermediate stage after the onset of a vaccination programme even before the post-vaccination stationary distribution has been reached. This is achieved by comparing the observed NVT disease reduction to the corresponding predicted reduction calculated under the assumption that the proportion of eliminated VT carriage *q* in the model equals the observed proportion of eliminated VT disease.

There are further assumptions that may pose limitations on the applicability of the proposed method. An identical degree of elimination and replacement in all age classes, i.e. the same values of *p* and *q* in equation (1) irrespective of age was assumed. In reality some differences across age classes may exist, but at least VT elimination has been observed to be nearly complete regardless of age class due to a strong herd effect. Of note, while full elimination in VT carriage is assumed, assumptions regarding vaccine efficacy against disease are irrelevant.

We assumed the long-term impact of vaccination on VT carriage is the same for all serotypes, i.e. complete or partial elimination. In addition, NVT carriage was assumed to be affected by vaccination only through replacement and vaccine-induced cross-protection was not included (for 6A, however, see Table S1). If differences between serotypes with respect to degree of elimination are assumed to exist, they could be taken into account in our model. Furthermore, as carriage data are typically available in terms of prevalences rather than incidences, also uncertainties regarding serotype specific differences in the duration of carriage affect the reliability of model predictions. Some serotypes are identified in carriage samples very rarely (e.g. serotypes 1 and 5). The current analysis suggests that the evaluation of rarely carried serotypes can rely solely on the disease incidence data.

Poor availability or reliability of serotype-specific carriage data across all age classes may limit the applicability of the model. In particular, the predictions are typically sensitive to assumptions regarding the VT carriage proportion. However, carriage data on the adult population are often sparse. Overestimating the VT proportion among adults may lead to underestimation of the effectiveness of vaccination. We demonstrated this by using two alternative VT carriage proportions (53% and 62% for PCV10) in the non-target population (individuals 5+ years of age) in the context of existing PCVs (Figure 3). In practice, the similarity of the VT proportion among children (68% UK vs. 62% in Finland for PCV10) suggests that the VT proportion among adults in the UK (53%) should be a good approximation to the corresponding proportion in Finland.

Several authors have previously discussed the importance of the invasiveness and the carriage incidences of the vaccine types relative to the non-vaccine types in assessing the net effectiveness of a vaccine [3], [4], [13]. Shea et al. [10] applied a replacement model similar to ours to predict the amount of pneumococcal acute otitis media following PCV13. Another line of related work is based on regression models utilising pre- and post-vaccination IPD incidences [11] or pre- and post-vaccination distributions of carriage [12]. Our results, however, appear to be the first to express the relationship between the invasiveness, the carriage incidences and the predicted IPD incidences in an operational manner that allows for the identification of optimal vaccine types in addition to calculation of their predicted effects if added to the vaccine composition. Moreover, our formulation enables an easy and explicit assessment of the role of age stratification in evaluating different PCV programmes.

Based on whole-genomic sequencing data, Croucher et al. [21] suggest that serotype replacement in carriage manifests itself within sequence clusters so that VT strains are replaced by related NVT strains while the cluster specific prevalences are largely unaffected by vaccination. Of note, taking into account within sequence cluster replacement of this type in our model is analogous to the way we have above handled replacement within age categories.

We have proposed tools for the quantification of the relative importance of individual pneumococcal serotypes in conjugate vaccine compositions under serotype replacement. Our examples used IPD data from Finland and carriage data from Finland and the UK. Contingent on the availability of data, our tools are easily applicable in other settings as well. However, in contrast to the relative succinctness of the underlying model, the data requirements for a successful application of the proposed tools are not straightforward to satisfy and underline the importance of the availability of age- and serotype-specific data on both pneumococcal carriage and disease.

## Supporting Information

Figure S1 **Pre- and post-vaccination (PCV7) serotype proportions in carriage in 3 locations.** For all data sets, only the nonvaccine type (NVT) carriage data were used and the proportions are calculated from the total number of NVT carriage isolates. Square roots of proportions are plotted to clarify presentation. Description of the data is given in panel E. In each panel (A–D), a linear relationship applies to the majority of the data, supporting the assumption of preserved serotype proportions after vaccination (assumption A1). A clear exception is 6A, which is likely attributable to 6A being a vaccine-related serotype for PCV7.
(PDF)

Figure S2 **Predicted reduction in IPD incidence as a function of pre-vaccination proportions of VT carriage and disease assuming partial replacement.** As in Figure 3, each curve corresponds to all possible combinations of the pre-vaccination proportions of VT carriage (x-axis) and VT disease (y-axis) that lead to the indicated level of reduction in IPD incidence (dashed curves). The results are shown for 50% (panel A) and 10% (panel B) replacement in carriage and for 12 scenarios corresponding to the age group of interest, <5 or 5+ year olds, vaccine composition (7, 10, 13 or 14 vaccine types, indicated by colour codes), and carriage data location, either Finland or the UK. In each case, the Finnish IPD data were used. For the 14-valent vaccine composition “Opt14p”, see Figure 5. For results under full replacement in carriage, see Figure 3. The pattern in Figures 3 and S2 illustrates how the predicted effects of various vaccine compositions under replacement depend on VT proportions in both IPD and carriage, with the importance of the IPD proportion becoming dominant as the degree of replacement approaches 0% (panel B).
(TIFF)

Figure S3 **Optimal serotype compositions assuming partial replacement.** Optimal serotype compositions were calculated for <5 year olds (panel A) and 5+ year olds (panel B) and for 11 scenarios corresponding to different degrees of replacement (0%, 10%, … 100%; x-axis). For each of the 11 scenarios, serotypes are listed from top to bottom in the order they are included in the optimal vaccine composition. The colour of each bar segment corresponds to serotype (colour codes as in Figure 2). The height of the bar segment corresponds to the additional annual IPD incidence eliminated due to the inclusion of the new vaccine type, calculated as a percentage of the pre vaccination total IPD incidence (i.e., 99 in panel A and 658 in B). The location of the bottom of each of the coloured bar segment indicates the proportion of IPD remaining (y-axis) after inclusion of the corresponding serotype in the vaccine composition consisting of the serotypes located above it. The yellow dashed line corresponds to 50% decrease in IPD if serotypes above it are included in the vaccine. For example, among the <5 year olds, assuming no replacement, a vaccine composed of the 4 best vaccine types (6B, 14, 19A and 23F) would result in a 64% decrease in IPD. Under full replacement, including the 4 best serotypes (14, 6B, 19A and 18C) would result in a 52% reduction.
(PDF)

File S1 **The program code with instructions and examples.**
(PDF)

File S2 **Details on assessing the effects of uncertainties in carriage proportions.**
(PDF)

Table S1 **Sensitivity of predictions on the assumed proportion of 6C carriage incidence.** Predicted annual IPD incidence under full replacement assuming different proportions (0%, 33% and 50%) of 6C carriage isolates among 6A/C carriage isolates, calculated for the vaccine compositions in Figure 5 (panel G). In addition, 2 compositions with serotype 6A as a vaccine related type are considered. The results in text correspond to 6C carriage proportion 33%. The table shows a moderate decrease in the predicted IPD incidence as the assumed 6C proportion increases.
(PDF)
