# Supplementary figures and images for: Optimal Serotype Compositions for Pneumococcal Conjugate Vaccination under Serotype Replacement
Source: PLoS Comput Biol. 2014 Feb 13;10(2):e1003477. doi: 10.1371/journal.pcbi.1003477 (PMC3923658; doi:10.1371/journal.pcbi.1003477)

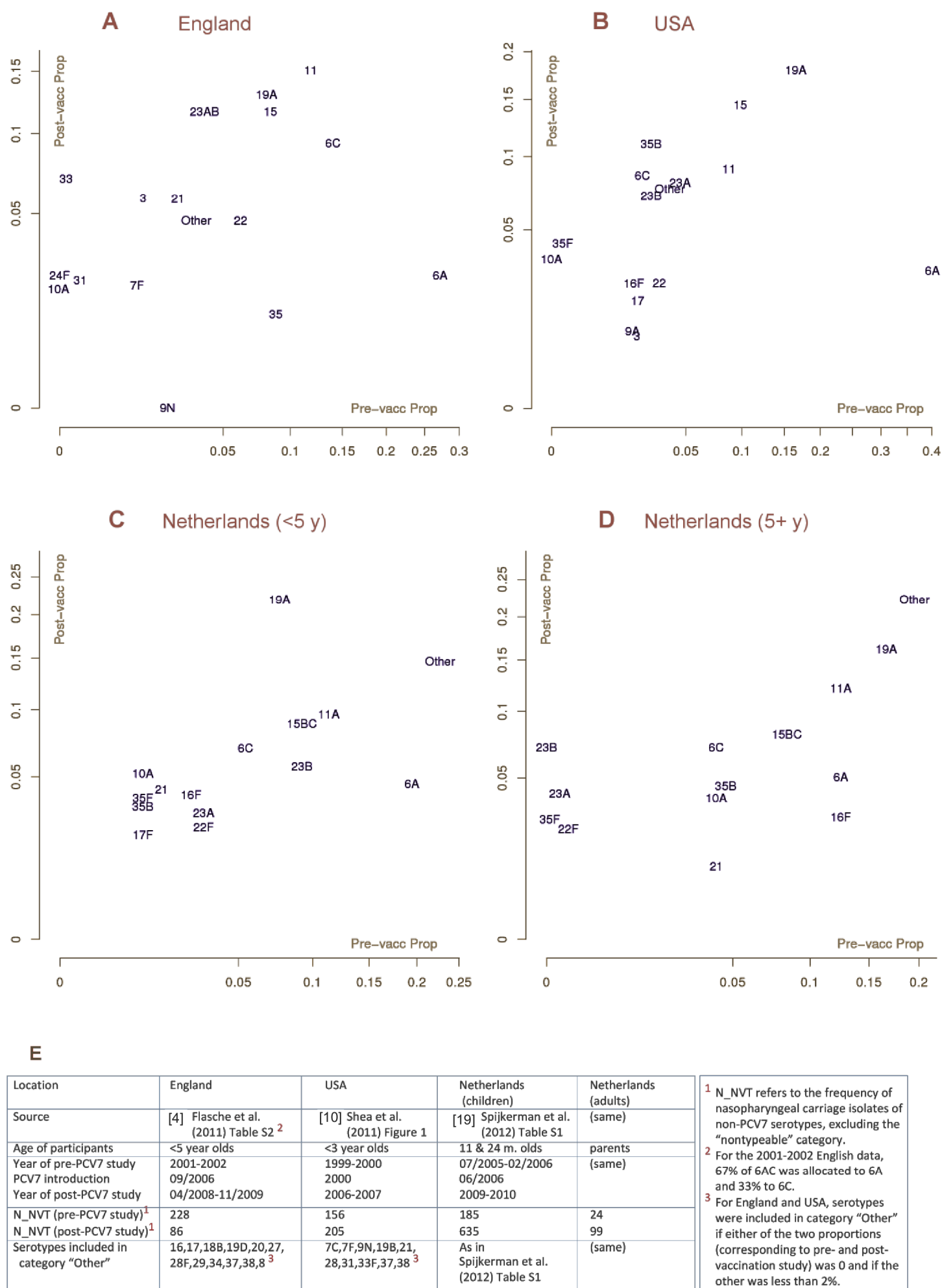

Figure S1

Supplement: Figure S1 — Pre- and post-vaccination (PCV7) serotype proportions in carriage in 3 locations. For all data sets, only the nonvaccine type (NVT) carriage data were used and the proportions are calculated from the total number of NVT carriage isolates. Square roots of proportions are plotted to clarify presentation. Description of the data is given in panel E. In each panel (A–D), a linear relationship applies to the majority of the data, supporting the assumption of preserved serotype proportions after vaccination (assumption A1). A clear exception is 6A, which is likely attributable to 6A being a vaccine-related serotype for PCV7. (PDF) [file pcbi.1003477.s001.pdf]

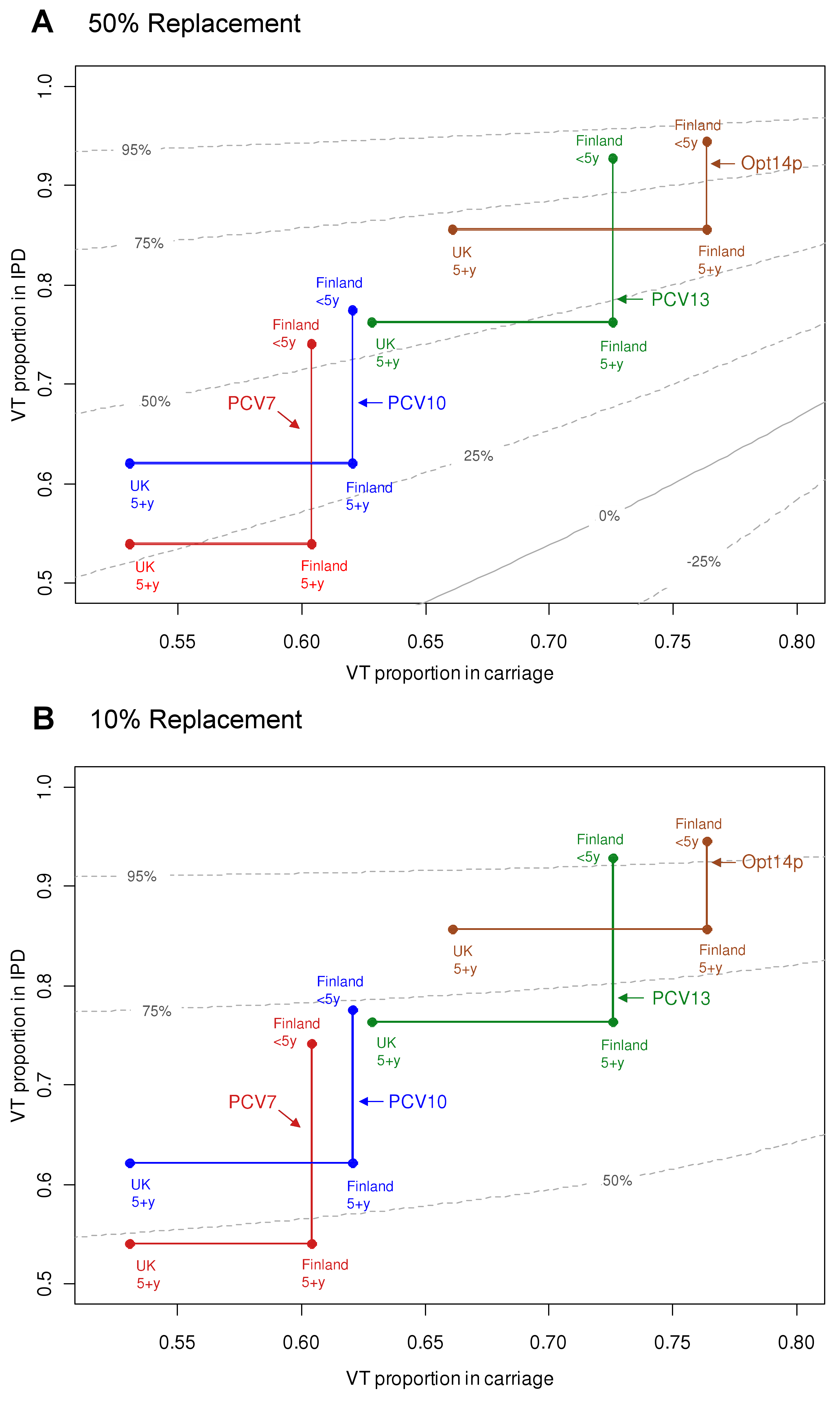

Supplement: Figure S2 — Predicted reduction in IPD incidence as a function of pre-vaccination proportions of VT carriage and disease assuming partial replacement. As in Figure 3, each curve corresponds to all possible combinations of the pre-vaccination proportions of VT carriage (x-axis) and VT disease (y-axis) that lead to the indicated level of reduction in IPD incidence (dashed curves). The results are shown for 50% (panel A) and 10% (panel B) replacement in carriage and for 12 scenarios corresponding to the age group of interest, <5 or 5+ year olds, vaccine composition (7, 10, 13 or 14 vaccine types, indicated by colour codes), and carriage data location, either Finland or the UK. In each case, the Finnish IPD data were used. For the 14-valent vaccine composition “Opt14p”, see Figure 5. For results under full replacement in carriage, see Figure 3. The pattern in Figures 3 and S2 illustrates how the predicted effects of various vaccine compositions under replacement depend on VT proportions in both IPD and carriage, with the importance of the IPD proportion becoming dominant as the degree of replacement approaches 0% (panel B). (TIFF) [file pcbi.1003477.s002.tiff]
