# Supplementary material for: Optimal Serotype Compositions for Pneumococcal Conjugate Vaccination under Serotype Replacement
Source: PLoS Comput Biol. 2014 Feb 13;10(2):e1003477. doi: 10.1371/journal.pcbi.1003477 (PMC3923658; doi:10.1371/journal.pcbi.1003477)
Supplement: File S1 — The program code with instructions and examples. (PDF) [file pcbi.1003477.s004.pdf]

File S1

The program code with instructions and examples.

S1.1 Data preparation

S1.2 Predicting the net effectiveness under a given vaccine composition

S1.3 Finding optimal vaccine serotype compositions

S1.4 The R-functions

This file contains a core set of functions implementing the basic tools presented in the manuscript and instructions on how to use the code. The code is written in the R programming language, which is freely available at www.r-project.org.

To run the examples in Sections S1.1-S1.3, first load the functions listed in Section S1.4. The calculation of the projected net effectiveness of a vaccination programme assuming a given vaccine composition is demonstrated in Section S1.2 and Section S1.3 shows how to use the code in searching for optimal vaccine compositions.

S1.1. Data preparation

In our example, we apply the code to the IPD data from Finland and carriage data from Finland and the UK. The data contain 24 serotypes and 2 age categories, the <5 and 5+ year olds.

```
## Read the annual IPD and carriage incidence data.
## The 0 entries in IPD and carriage data are replaced by small values.

serotypes<-c(
    "19F",   "23F",   "6B",   "14",    "9V",    "4",     "18C",   "1",     "7",
    "6A",    "19A",   "3",    "8",     "9N",    "10",    "11",    "12",    "15",
    "16",    "20",    "22",   "23A",  "33",    "35",   "38",    "6C",    "Oth")
car_under5<-c(
  156030,   156030,   126990,  41200,  22290,  12830,  10130,     10,  14180,
   54940,    24320,    12160,   1350,  20940,   4050,  72270,     10,  33100,
    3380,     1350,    12160,   3380,    680,  30400,   4050,  27470,  24320 )
car_over5<-c(
  168100,  314800,  256700,  209800, 114100,  62500,  200700,     100,    100,
  158800,  54900,    30800,    8800,   8800,  20800,   97700,     100,    100,
  191900,  25200,    72500,   22000,    100,  71300,     100,  79400,  330100 )
ipd_under5<-c(
  7.78,    7.88,   24.39,   20.76,    2.91,    2.91,    6.64,    0.31,    3.02,
  3.94,    9.88,    1.25,    0.10,    0.83,    0.41,    0.42,    0.21,    1.98,
  0.21,    0.01,    0.93,    0.10,    0.42,    0.31,    0.42,    0.01,    0.73 )
ipd_over5<-c(
   28.51,   53.72,   29.53,   99.43,   43.07,   76.99,   24.39,    6.58,   46.88,
   17.42,   20.54,   55.04,   11.21,   25.20,    6.28,   12.76,   13.89,    9.18,
    4.73,    3.29,   29.03,    4.40,    5.64,   12.41,    1.43,    5.50,   11.20 )
```

```
## Combine the data into 2 matrices of dimension 27*2:
IPD<-cbind(ipd_under5, ipd_over5)
Car<-cbind(car_under5, car_over5)

## Row numbers corresponding to the 3 different PCV formulations
## in matrices IPD and Car. Note: there is no serotype 5 in our data.
pcv7rows<-seq(7); pcv10rows<-seq(9); pcv13rows<-seq(12)
```

S1.2 Predicting the net effectiveness under a given vaccine composition

The function *Vaccination* is an implementation of equation (1). Its arguments are the pre-vaccination IPD and carriage incidences by age category and serotype. It returns the predicted post-vaccination versions of these two matrices. If only one age category is considered, the arguments can be vectors.

```
## Example S1.2A: Calculate the predicted incidence of IPD for the non-vaccine
## types(NVTs) under PCV13. The predictions are calculated separately for the
## two age classes. These are the values reported on the bottom panel in
## Figure 2 (there given as per 100K incidences).

postvacc <-Vaccination(IPD,Car,VT_rows=pcv13rows,p=1,q=1)
cbind(serotypes,postvacc[[1]])

serotypes     8    9N   10   11   12   15   16   20  22   23A  33   35   38  6C   Oth
ipd_under5    0.4  3    1.5  1.5  0.8  7.2  0.8  0   3.4  0.4  1.5  1.1  1.5 0    2.7
ipd_over5     30.2 67.8 16.9 34.3 37.4 24.7 12.7 8.9 78.1 11.8 15.2 33.4 3.8 14.8 30.1
```

```
## Example S1.2B: Decrease in IPD incidence after adding a single new serotype
## to PCV13 separately for the two age categories.

next_under5<-NextVT(IPD[,1],Car[,1], VT_rows=pcv13rows,p=1)
next_over5 <-NextVT(IPD[,2],Car[,2], VT_rows=pcv13rows,p=1)
rbind(serotypes, next_under5, next_over5)

serotypes     8    9N   10  11    12   15   16    20   22   23A 33   35   38  6C
ipd_under5    0.2  0.8  1.1 -8.9  0.8  4.2  0.4   0    2.2  0   1.5  -2.4 1.1 -3.2
ipd_over5     26.4 64.5 7.7 -10.9 37.3 24.7 -93.3 -2.5 49.2 1.9 15.1 1.2  3.8 -23
```

S1.3. Finding optimal vaccine serotype compositions

The search for optimal vaccine compositions is implemented in the function “OptimalSequence”. The optimum is found for the entire data, i.e. taking into account all age categories (columns) included in the matrix arguments. If optimum is intended to be found only for one age category, the arguments for “OptimalSequence” should be vectors corresponding to this category.

```
## Example S1.3A: The optimal sequence for under 5 year olds when replacement is 100%.
## The output shows the decreases in IPD incidence for each step,
## corresponding to Figure 5(C). The last serotype (row 27, the category "Other")
## is excluded from any vaccine composition but is taken into account as a
## replacing serotype at each stage.

opt<-OptimalSequence(IPD[,1],Car[,1],VT_rows=0,Excluded_rows=27,p=1.0,HowmanyAdded=20)
rbind(serotypes[opt[1,]],opt[2,])

rank  1    2    3    4   5   6   7   8   9   10  11  12  13  14  15  16  17  18  19  20
stype 14   6B   19A  18C 7   4   9V  6A  23F 19F 15  3   22  33  9N  38  10  1   12  16
eff  16.8 15.4 10.3 7.8 2.9 3  2.5 2.2 2.4 5.1 3.3 3.7 2.7 1.8 1.9 1.7 1.8 1.6 1.1 0.9


## Example S1.3B: The optimal sequence for the whole population when
## replacement is 50% and the current composition includes the PCV7 serotypes.

opt<-OptimalSequence(IPD,Car, VT_rows=pcv7rows,Excluded_rows=length(serotypes),
p=0.5,HowmanyAdded=17)
rbind(serotypes[opt[1,]],opt[2,])

rank     1    2    3    4    5    6   7    8    9    10   11   12   13   14   15  16  17
stype    3    7    19A  9N   22   12  6A   15   8    35   11   1    33   10   23A 6C  20
effect 82.3 79.3 42.8 40.9 41.6 24 21.1 20.8 20.3 17.7 15.2 15.1 13.6 13.7 8.6 6.1 6.5
```

S1.4. The R-functions

```
################################################################################
##
##  R code for the core methods introduced in
##     Markku Nurhonen and Kari Auranen:
##     "Optimal serotype compositions for pneumococcal conjugate
##      vaccination under serotype replacement",
##     PLoS Computational Biology, 2014.
##
################################################################################

## List of arguments common to most functions:
##
## IPD = matrix of IPD incidences by age class (columns) and serotype (rows)
## Car = corresponding matrix of carriage incidences
## VT_rows = vector of the row numbers in matrices IPD and Car
##           corresponding to vaccine types (VT_rows=0 for no vaccination)
## p = proportion of lost VT carriage which is replaced by NVT carriage
## q = proportion of VT carriage lost either due to elimination or replacement
##
## This code includes 4 functions:
## Vaccination, NextVT, OptimalSequence and OptimalVacc.
##
```

```
Vaccination<-function(IPD,Car,VT_rows,p,q)
##
## Result:
## A list of 2 matrices: IPD and carriage incidences
## after vaccination (corresponding to matrices IPD and Car).
##                                  [Markku Nurhonen 2013]
##
{if (VT_rows[1]>0)
{IPD<-as.matrix(IPD); Car<-as.matrix(Car)
 # Post vaccination carriage incidences
   Car_Total<-t(matrix(apply(Car,2,sum),dim(Car)[2],dim(Car)[1]))
   Car2<-Car; Car2[VT_rows,]<-0
   Car_NVT<-t(matrix(apply(Car2,2,sum),dim(Car2)[2],dim(Car2)[1]))
   Car_VT<-Car_Total-Car_NVT
   CarNew<-q*(1+p*Car_VT/Car_NVT)*Car2+(1-q)*Car
 # Post vaccination IPD incidences
   NVT_rows<-seq(dim(IPD)[1])[-1*VT_rows]
   # CCR=Case-to-carrier ratios
   CCR<-IPD/Car ;  IPDNew<-0*IPD
   # Apply the equation appearing above
   # equation (1) in text for each serotype.
   # First term applies to NVTs.
   IPDNew[VT_rows,]<-(1-q)*IPD[VT_rows,]
   # Second term applies to NVTs.
   IPDNew[NVT_rows,]<-((Car_NVT+p*q*Car_VT)*(Car/Car_NVT)*CCR)[NVT_rows,]}
 else {IPDNew<-IPD; CarNew<-Car}
 list(IPDNew,CarNew) }


NextVT<-function(IPD,Car,VT_rows,p)
##
## Result:
## A vector of decreases in IPD due to adding a serotype
## to the vaccine. If VT_rows=0, initially no vaccination.
## For row indexes incuded in VT_rows, the result is 0.
##                                 [Markku Nurhonen 2013]
##
{ IPD<-as.matrix(IPD); Car<-as.matrix(Car)
 ## VaccMat = IPD and Car matrices after vaccination
  VaccMat<-Vaccination(IPD,Car,VT_rows,p,1)
  IPD<-VaccMat[[1]]; Car<-VaccMat[[2]]
 ## Total_IPD,Total_Car = Matrices corresponding to
 ## overall IPD and carriage in each age class.
  Total_IPD<-t(matrix(apply(IPD,2,sum),dim(IPD)[2],dim(IPD)[1]))
  Total_Car<-t(matrix(apply(Car,2,sum),dim(Car)[2],dim(Car)[1]))
 ## Effect = decrease in IPD when one serotype is added to the vaccine.
 ## See equation (3) in text.
  Effect<-(Total_IPD-IPD)*((IPD/(Total_IPD-IPD))-(p*Car/(Total_Car-Car)))
 ## Special case when only one NVT remains.
  IPD_nonzero<-which(apply(IPD,1,sum)!=0)
  if (length(IPD_nonzero)==1) {Effect[IPD_nonzero,]<-IPD[IPD_nonzero,]}
 ## Result is obtained after summation over age classes.
  apply(Effect,1,sum) }
```

```
OptimalSequence<-function(IPD,Car,VT_rows,Excluded_rows,p,HowmanyAdded)
##
## Starting from VTs indicated by the vector VT_rows
## (VT_rows=0, for no vaccination) sequentially add new VTs
## to the vaccine composition s.t. at each step the optimal
## serotype (corresponding to largest decrease in IPD) is added.
##
## Excluded_rows = Vector of indexes of the rows in matrices
##     IPD and Car corresponding to serotypes that are not to
##     be included in a vaccine composition, e.g. a row
##     corresponding to a group of serotypes labelled "Other".
##     Enter Excluded_rows=0 for no excluded serotypes.
## HowmanyAdded = number of VTs to be added.
##
## Result:
## Matrix of dimension 2*HowmanyAdded with 1st row indicating
## the row numbers of added serotypes in the order they appear
## in the sequence. The 2nd row lists the decreases in IPD
## due to addition of each type.    [Markku Nurhonen 2013]
##
{ IPD<-as.matrix(IPD); Car<-as.matrix(Car)
  ## First check the maximum possible number of added VTs.
  VT_howmany<-length(VT_rows)
  if (VT_rows[1]==0) {VT_howmany<-0}
  Excluded_howmany<-length(Excluded_rows)
  if (Excluded_rows[1]==0) {Excluded_howmany<-0}
  HowmanyAdded<-min(HowmanyAdded,dim(IPD)[1]-(VT_howmany+Excluded_howmany))
  BestVTs<-BestEffects<-rep(0,HowmanyAdded)
  ## Sequential procedure: at each step find the best additional VT.
  for (i in 1:HowmanyAdded)
  ## Effects = Decrease in IPD after addition of each serotype
   {Effects<-NextVT(IPD,Car,VT_rows,p)
  ## Set Effects for VTs and excluded types equal to small values
  ## so that none of these will be selected as the next VT.
    minvalue<- -2*max(abs(Effects))
    if (Excluded_howmany>0) {Effects[Excluded_rows]<-minvalue}
    if (VT_rows[1]>0)       {Effects[VT_rows]<-minvalue}
  ## BestVTs[i] = Index of serotype with maximum decrease in IPD.
    BestVTs[i]<-order(-1*Effects)[1]
  ## BestEffects[i] = Decrese in IPD due to addition of BestVTs[i]
  ##                  to the vaccine.
    BestEffects[i]<-Effects[BestVTs[i]]
    VT_rows<-c(VT_rows,BestVTs[i])
    if (VT_rows[1]==0) {VT_rows<-VT_rows[-1]}
    VaccMat<-Vaccination(IPD,Car,VT_rows,p,1)
    IPD<-VaccMat[[1]]; Car<-VaccMat[[2]] }
   t(matrix(c(BestVTs,BestEffects),HowmanyAdded,2)) }

OptimalVacc<-function(IPD,Car,VT_rows,p,q,HowmanyAdded)
##
## Result:
## A list of 3 elements: (1) Row numbers of serotypes in the optimal
## vaccine composition (2)-(3) IPD and carriage incidences
## by serotype and age class corresponding to the optimal
## vaccine formed using the sequential procedure in the
## function OptimalSequence.     [Markku Nurhonen 2013]
##
{ Additional_VTs<-OptimalSequence(IPD,Car,VT_rows,p,HowmanyAdded)[1,]
  All_VTs<-c(VT_rows,Additional_VTs)
  if (All_VTs[1]==0) All_VTs<-All_VTs[-1]
  VaccMat<-Vaccination(IPD,Car,All_VTs,p,q)
  list(All_VTs,VaccMat[[1]],VaccMat[[2]]) }
## (end of R code)
```
