# Supplementary material for: Optimal Serotype Compositions for Pneumococcal Conjugate Vaccination under Serotype Replacement
Source: PLoS Comput Biol. 2014 Feb 13;10(2):e1003477. doi: 10.1371/journal.pcbi.1003477 (PMC3923658; doi:10.1371/journal.pcbi.1003477)

File S2

Details on assessing the effects of uncertainties in carriage proportions.

Uncertainties in carriage proportions

We obtained serotype proportions in pneumococcal carriage from 2 data sets. The <5 year old proportions were obtained from a Finnish data set and the adult proportions from a UK data set (see Methods). These data can be assumed to identify the most common serotypes in carriage, but, for the purposes of our model, they are lacking in accuracy for several reasons. The sources of uncertainty in the carriage data can be summarised as follows:

(1) Sampling. The serotype proportions are based on 1288 (Finland) and 245 (UK) samples of nasopharyngeal carriage. Accordingly, the observed data contain some statistical sampling error.

(2) Age. The data were collected from individuals whose age does not always match the age of the population segment to which the data are applied in the model.

(3) Location. The data were collected from specific locations in Finland and UK and for this reason the data may not reflect the full diversity of serotype carriage at a national level.

(4) Time. The data were collected at a specific point in time and may reflect secular trends.

Data on IPD

The IPD data used in this paper was very substantial and included all reported IPD cases in Finland during 2000-2009 (993 among the <5 year old population and 6554 among the 5+ year old population). This means that statistical uncertainty in the serotype distribution in IPD is much smaller than that in carriage. Moreover, because the IPD data covers 10 pre-vaccination years, the estimated serotype distribution can be expected to even out secular fluctuations. For these reasons, we do not consider sensitivity of results with respect to uncertainties in IPD data.

Implications of uncertainties in carriage

Sensitivity analysis under alternative serotype proportions in carriage affects mostly the role of those serotypes that are at least moderately common in both IPD and carriage. Assuming that data on IPD is accurate, then if there is a chance that the actual carriage of such a serotype is much less carried than suggested by the data, inclusion of this serotype in the vaccine composition may be in order as a precaution even if the observed data would not favour its inclusion (cf. 19F among adults in Figure 5). On the other hand, if a serotype which is at least moderately common in IPD is in reality much more common in carriage than suggested by the data, the effectiveness of a vaccine composition where this serotype is included may be less than predicted by the data (cf. 22 among adults in Figure 5).

■ Methods for assessing the effects of uncertainties in carriage

The Dirichlet distribution is a standard multivariate probability distribution for a set of random variables that sum up to unity. It is commonly used in Bayesian statistical analysis as a prior distribution for a set of probabilities. In particular, according to standard results, if the prior distribution for a set of probabilities in a multinomial model is Dirichlet, also the posterior distribution is a Dirichlet distribution. In our data, this posterior can be used in sensitivity studies to account for sampling errors. However, the resulting posterior distribution may be far too narrow to be useful as a distribution accounting for uncertainties in serotype proportions as most of these uncertainties are typically related to reasons other than the sampling errors (i.e. reasons 2-4 above). This applies for example to the Finnish carriage data with large sample sizes. Hence, in our sensitivity studies we used a subjectively chosen Dirichlet distribution, which is more sparse than what would be obtained as a posterior distribution based on accounting for the uncertainties in sampling errors alone.

The distribution we applied in Figure 5 is a Dirichlet distribution with parameter vector 60p, where p is the vector of observed serotype proportions. The level of uncertainty in individual serotype proportions induced by this distribution is illustrated in Figure S2.1 below. For example, for a serotype proportion at 0.15, 70% of the perturbed proportions are between 0.1 and 0.2. For a proportion 0.02, 35% of the perturbed proportions are below 0.01.

Figure S2.1. Illustration of the Dirichlet distribution applied to form the perturbed serotype proportions in Figure 5. Distributions are shown corresponding to original serotype proportions at 20%, 15%, 10%, and 5% in panel A; 1% and 2% in panel B. The colour codes are the same as in Figure 5 and correspond to the percentiles of the distribution as printed in blue colour on top of the first bar. Note the different scale (x-axis) in panels A and B.

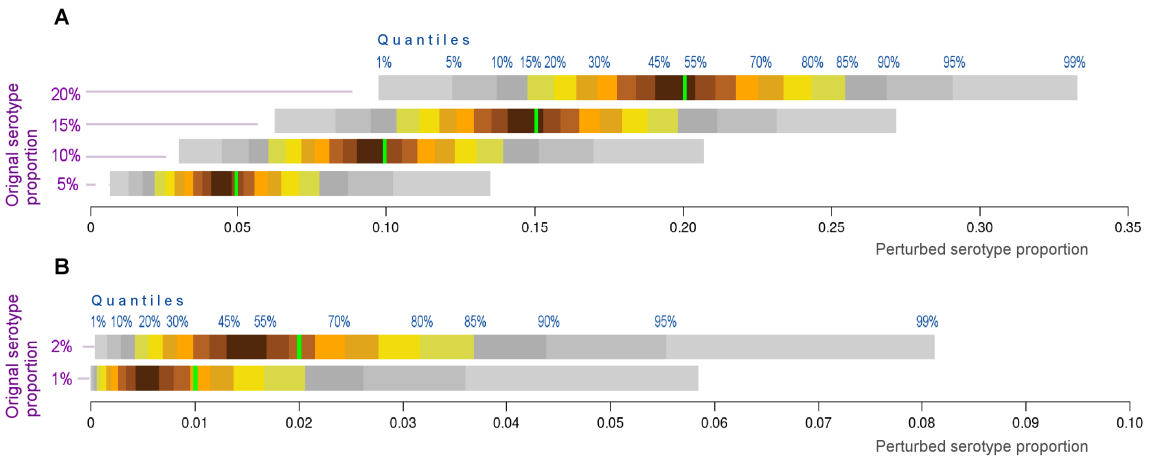

Supplement: File S2 — Details on assessing the effects of uncertainties in carriage proportions. (PDF) [file pcbi.1003477.s005.pdf]
