# Supplementary material for: Optimal Serotype Compositions for Pneumococcal Conjugate Vaccination under Serotype Replacement
Source: PLoS Comput Biol. 2014 Feb 13;10(2):e1003477. doi: 10.1371/journal.pcbi.1003477 (PMC3923658; doi:10.1371/journal.pcbi.1003477)
Supplement: Table S1 — Sensitivity of predictions on the assumed proportion of 6C carriage incidence. Predicted annual IPD incidence under full replacement assuming different proportions (0%, 33% and 50%) of 6C carriage isolates among 6A/C carriage isolates, calculated for the vaccine compositions in Figure 5 (panel G). In addition, 2 compositions with serotype 6A as a vaccine related type are considered. The results in text correspond to 6C carriage proportion 33%. The table shows a moderate decrease in the predicted IPD incidence as the assumed 6C proportion increases. (PDF) [file pcbi.1003477.s006.pdf]

	<5 year olds			5+ year olds		
6C carriage p.[1]	0	33%	50%	0	33%	50%
6C IPD prop. [2]	0	0	0	0	24%	24%
No Vaccination		99			658	
PCV7		64			645	
PCV7+6A[3]	71	65	62	748	703	676
PCV10		58			531	
PCV10+6A[3]	64	58	55	605	571	550
PCV13	29	26	24	443	420	403
Opt7c		40			496	
Opt10c	34	30	28	603	567	545
Opt13c	23	20	19	383	364	347
Opt7a		78			353	
Opt10a		76			298	
Opt13a		92			250	
Opt14p	26	23	22	290	279	267
Vac15	25	22	21	373	355	339
Rank(6A/C)[4]	11	-	-	18	-	-
Rank(6A)[5]	-	8	7	-	18	14
Rank(6C)[5]	-	25	26	-	23	24

(1) Proportion of 6C carriage among 6A/C
(2) Among the IPD samples, when 6C was distinguished from 6A, the proportion of 6C among 6A/C was 0% in the <5 year old and 24% in the 5+ year old age category (see text).
(3) 6A included as a possible vaccine related serotype
(4) Rank of 6A/C among the 25 serotypes
(5) Rank of 6A and 6C among the 26 serotypes when considered separately

Blank entries correspond to vaccine compositions not including 6A or 6C (results not affected by assumed 6C proportion).

Table S1
