# Supplementary material for: Optimal Serotype Compositions for Pneumococcal Conjugate Vaccination under Serotype Replacement
Source: PLoS Comput Biol. 2014 Feb 13;10(2):e1003477. doi: 10.1371/journal.pcbi.1003477 (PMC3923658; doi:10.1371/journal.pcbi.1003477)
Supplement: Figure S3 — Optimal serotype compositions assuming partial replacement. Optimal serotype compositions were calculated for <5 year olds (panel A) and 5+ year olds (panel B) and for 11 scenarios corresponding to different degrees of replacement (0%, 10%, … 100%; x-axis). For each of the 11 scenarios, serotypes are listed from top to bottom in the order they are included in the optimal vaccine composition. The colour of each bar segment corresponds to serotype (colour codes as in Figure 2). The height of the bar segment corresponds to the additional annual IPD incidence eliminated due to the inclusion of the new vaccine type, calculated as a percentage of the pre vaccination total IPD incidence (i.e., 99 in panel A and 658 in B). The location of the bottom of each of the coloured bar segment indicates the proportion of IPD remaining (y-axis) after inclusion of the corresponding serotype in the vaccine composition consisting of the serotypes located above it. The yellow dashed line corresponds to 50% decrease in IPD if serotypes above it are included in the vaccine. For example, among the <5 year olds, assuming no replacement, a vaccine composed of the 4 best vaccine types (6B, 14, 19A and 23F) would result in a 64% decrease in IPD. Under full replacement, including the 4 best serotypes (14, 6B, 19A and 18C) would result in a 52% reduction. (PDF) [file pcbi.1003477.s003.pdf]

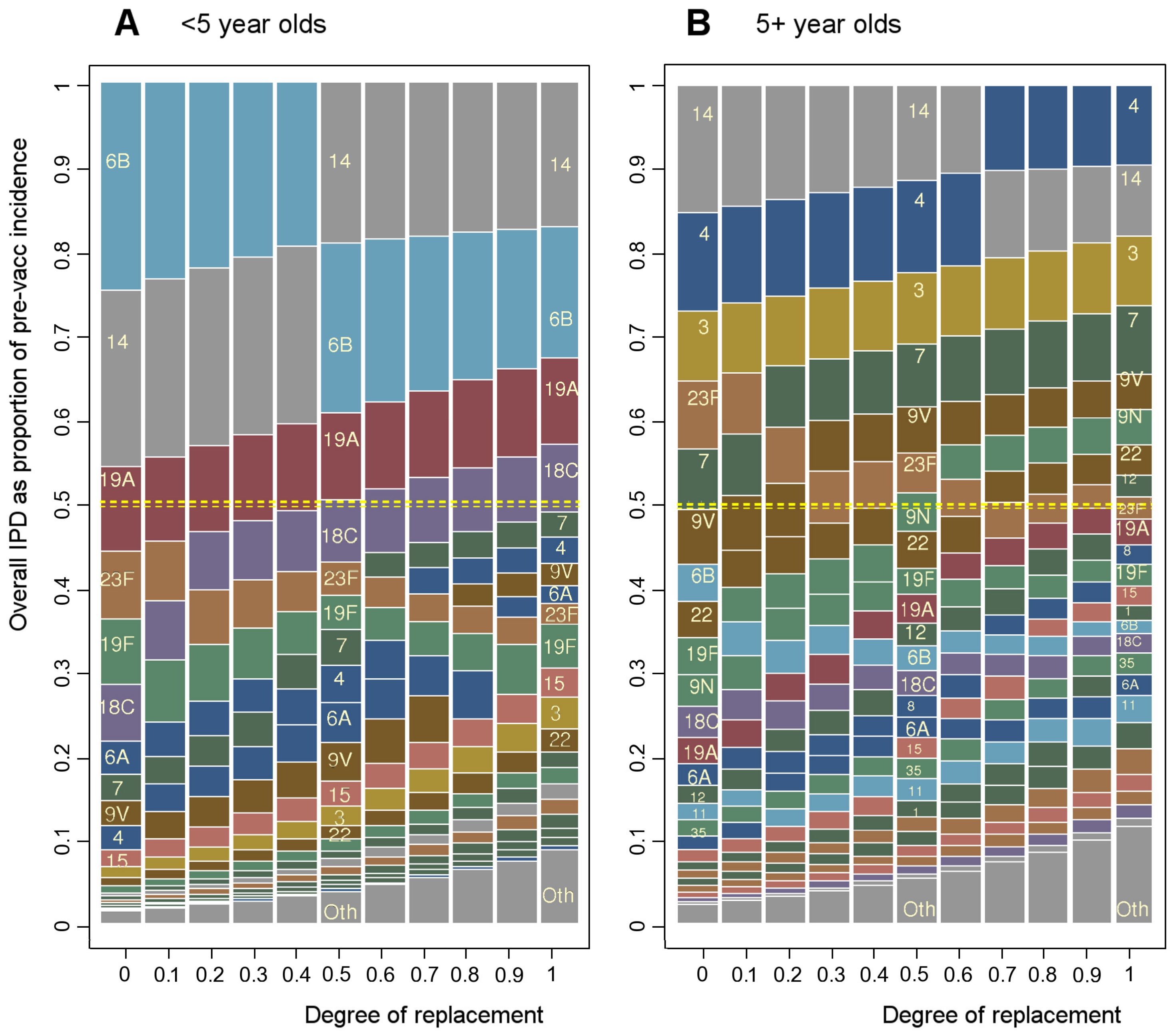

A
<5 year olds
B
5+ year olds
Overall IPD as proportion of pre-vacc incidence
0
0.1
0.2
0.3
0.4
0.5
0.6
0.7
0.8
0.9
1
Degree of replacement
Degree of replacement

Figure S3
